# AI-assisted differentiation of nontuberculous mycobacterial pulmonary disease from colonization: a multi-center study

**DOI:** 10.1186/s13244-025-02131-1

**Published:** 2025-11-09

**Authors:** Chia-Jung Liu, Yueh-Chun Liu, Yu-Hsuan Chen, Yu-Sen Huang, Po-Chih Kuo, Meng-Rui Lee, Lu-Cheng Kuo, Jann-Yuan Wang, Chao-Chi Ho, Jin-Yuan Shih, Chong-Jen Yu

**Affiliations:** 1 Department of Internal Medicine, National Taiwan University Hospital, Hsin-Chu Branch, Hsinchu, Taiwan; 2 Graduate Institute of Clinical Medicine, College of Medicine, National Taiwan University, Taipei, Taiwan; 3 Department of Computer Science, National Tsing Hua University, Hsinchu, Taiwan; 4 Department of Internal Medicine, Min-Sheng General Hospital, Taoyuan, Taiwan; 5 Department of Medical Imaging, National Taiwan University Hospital, Taipei, Taiwan; 6 Department of Internal Medicine, National Taiwan University Hospital, Taipei, Taiwan

**Keywords:** Nontuberculous mycobacteria, Colonization, Deep learning, Artificial intelligence

## Abstract

**Objectives:**

Differentiating between nontuberculous mycobacteria (NTM) pulmonary disease (NTM-PD) and colonization (NTM-PC) is clinically important but difficult. It remains unknown whether artificial intelligence utilizing clinical data and chest CT images could address this clinical problem.

**Materials and methods:**

Patients were retrospectively recruited with NTM isolation from respiratory specimens in two hospitals. Their disease or colonization status was determined by three NTM experts. We developed a multimodal deep learning model named NTMNet, which integrates chest CT scans and clinical data (including age, sex, acid-fast smear [AFS] results, and mycobacterial species) to predict NTM disease status. The performance of NTMNet was evaluated on both internal and external test sets.

**Results:**

A total of 324 NTM-PC patients and 285 NTM-PD patients were included. Among the internal and external test sets, the area under the receiver operating characteristic curve (AUC) for predicting NTM disease status using CT imaging was 0.73 (95% CI: 0.62–0.82) and 0.78 (95% CI: 0.75–0.83), respectively. When imaging data were integrated with clinical information, our NTMNet model achieved AUC values of 0.85 (95% CI: 0.80–0.93) and 0.82 (95% CI: 0.78–0.89), respectively. Furthermore, our NTMNet model demonstrated comparable accuracy to that of three experienced pulmonologists in determining NTM disease status in the reader study.

**Conclusion:**

Our multimodal NTMNet exhibited satisfactory performance in distinguishing disease status among patients with respiratory NTM isolates. This deep learning-based model has the potential to assist physicians in clinical management, achieving diagnostic accuracy comparable to that of pulmonologists.

**Critical relevance statement:**

A deep learning model leveraging chest computed tomography images and clinical data effectively differentiated NTM disease status, achieving a classification accuracy comparable to that of pulmonologists and demonstrating its potential to support accurate NTM diagnosis in clinical settings.

**Key Points:**

- Accurately distinguishing nontuberculous mycobacteria (NTM) disease status is clinically important but challenging.
- The NTMNet model effectively differentiated the NTM disease status and matched the performance of the pulmonologists.
- The NTMNet model could be a potential diagnostic tool for patients with respiratory NTM isolates.

**Graphical Abstract:**

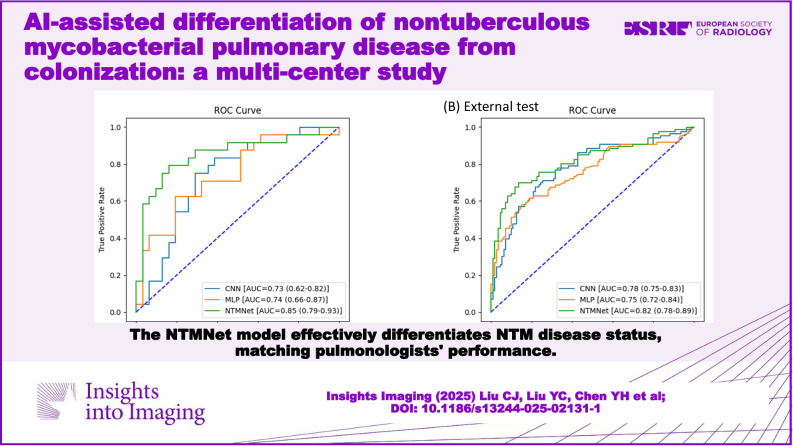

## Introduction

Notably, the detection of nontuberculous mycobacteria (NTM) in respiratory specimens can indicate either NTM pulmonary disease (NTM-PD) or NTM pulmonary colonization (NTM-PC) [1]. Timely differentiation between NTM-PD and NTM-PC is crucial because management differs between these two conditions. Antimycobacterial treatment is unnecessary for patients with NTM-PC [2]. Conversely, for those diagnosed with NTM-PD, a comprehensive approach, including pulmonary rehabilitation programs, optimal nutritional strategies, and possibly antimicrobial treatments, should be carefully considered [3, 4].

The standard pharmacological treatment for NTM-PD involves a lengthy and complex multidrug antimicrobial regimen that is often associated with multiple side effects [5]. Incorrectly diagnosing NTM-PC patients as NTM-PD could result in unnecessary treatment and expose patients to treatment-related side effects. Conversely, misdiagnosing NTM-PD patients as NTM-PC may lead to delayed diagnosis, resulting in increased disease severity and functional loss [6].

The current NTM guideline recommends that clinicians integrate clinical, radiographic, and microbiological data to distinguish between NTM-PD and NTM-PC [4]. Nevertheless, determining the clinical status of NTM is often challenging. For instance, it can be perplexing to differentiate between patients with an active NTM infection and those with structural lung disease exhibiting NTM colonization only [7]. To address this clinical challenge, studies have been conducted to assess the differences between NTM-PD and NTM-PC. These findings suggest that female sex, a low body mass index, a history of previous pulmonary tuberculosis (TB), comorbidities such as chronic obstructive pulmonary disease, a positive sputum acid-fast smear (AFS), isolation of *Mycobacterium kansasii* from respiratory tract samples, and radiographic findings indicating nodular bronchiectasis and cavitary lesions are factors predictive of NTM-PD rather than NTM-PC [8, 9]. Despite these efforts, a single definitive and objective test to differentiate NTM-PD from NTM-PC is still lacking.

Given the recent rapid evolution of artificial intelligence (AI), deep learning models in neural networks have shown promising results in distinguishing between NTM-PD and pulmonary TB based on chest X-ray (CXR) and chest computed tomography (CT) [10, 11]. Furthermore, another study demonstrated that a deep learning model may be able to predict the long-term mortality of patients with NTM-PD using CXRs, with its accuracy increasing when CXRs were combined with clinical information [12]. These findings provide evidence that AI models have the potential to recognize image features and clinical information associated with NTM-PD. Therefore, we hypothesize that AI might offer a solution for distinguishing between NTM-PD and NTM-PC.

Hence, we initiated this study to evaluate whether a deep learning model using clinical information and chest CT could be employed to differentiate between NTM-PD and NTM-PC among patients with NTM isolation from respiratory specimens.

## Materials and methods

### Study design

This study was conducted at two hospitals, one serving as the internal cohort and the other serving as the external cohort. The institutional review board of National Taiwan University Hospital approved this study (111-177-F). The objective was to investigate the performance of the deep learning model in distinguishing between NTM-PD and NTM-PC. We retrospectively identified adult patients (> 20 years old) with NTM isolated from respiratory specimens and a chest CT performed within 3 months of the NTM isolation between January 2017 and December 2021. Considering the prevalence and clinical significance of the known NTM species [13], we enrolled only patients with isolates of the *Mycobacterium avium* complex*, M. abscessus* complex, and *M. kansasii* in this study.

Three clinical experts, each with more than 5 years of clinical experience in managing patients and conducting research in the NTM field, independently evaluated the NTM disease status of the enrolled participants. Their assessments adhered to the current NTM-PD guidelines [4]. Owing to our study’s retrospective nature and the lack of standardized NTM surveillance, clinical experts were instructed not to consider the number of NTM samples collected and cultured when they made their judgments. In the event of discrepancies among the three experts (nonconsensual status), the NTM disease status was determined using a majority rule approach. Owing to the demanding workload involved in interpreting chest CT images by the three experts, the study included approximately 60% of all cases with available chest CT scans at the participating hospital during the study period.

### Dataset establishment and data collection

The patients enrolled in the internal cohort were assigned randomly to the training, validation, and test sets using fixed ratios of 70%, 15%, and 15%, respectively. All patients in the external cohort were exclusively assigned to the external test set. Additionally, to ensure clarity in evaluating the performance of the deep learning model, only patients with a consensus on disease status by all three clinical experts were included in both the internal and external test sets. All the images were standardized through the preprocessing procedure as described in Appendix A.

We collected the following data from the enrolled patients: age; sex; microbiological reports, including AFS and NTM species; and chest CT images and radiographic patterns (fibrocalcified lesions, nodules or masses, consolidation, cavitation, bronchiectasis, and pleural effusion). The time interval between the selected chest CT image and the date of respiratory specimen collection for NTM isolation was restricted to 3 months.

### Development of the deep learning model—NTMNet

**Fig. 1:**
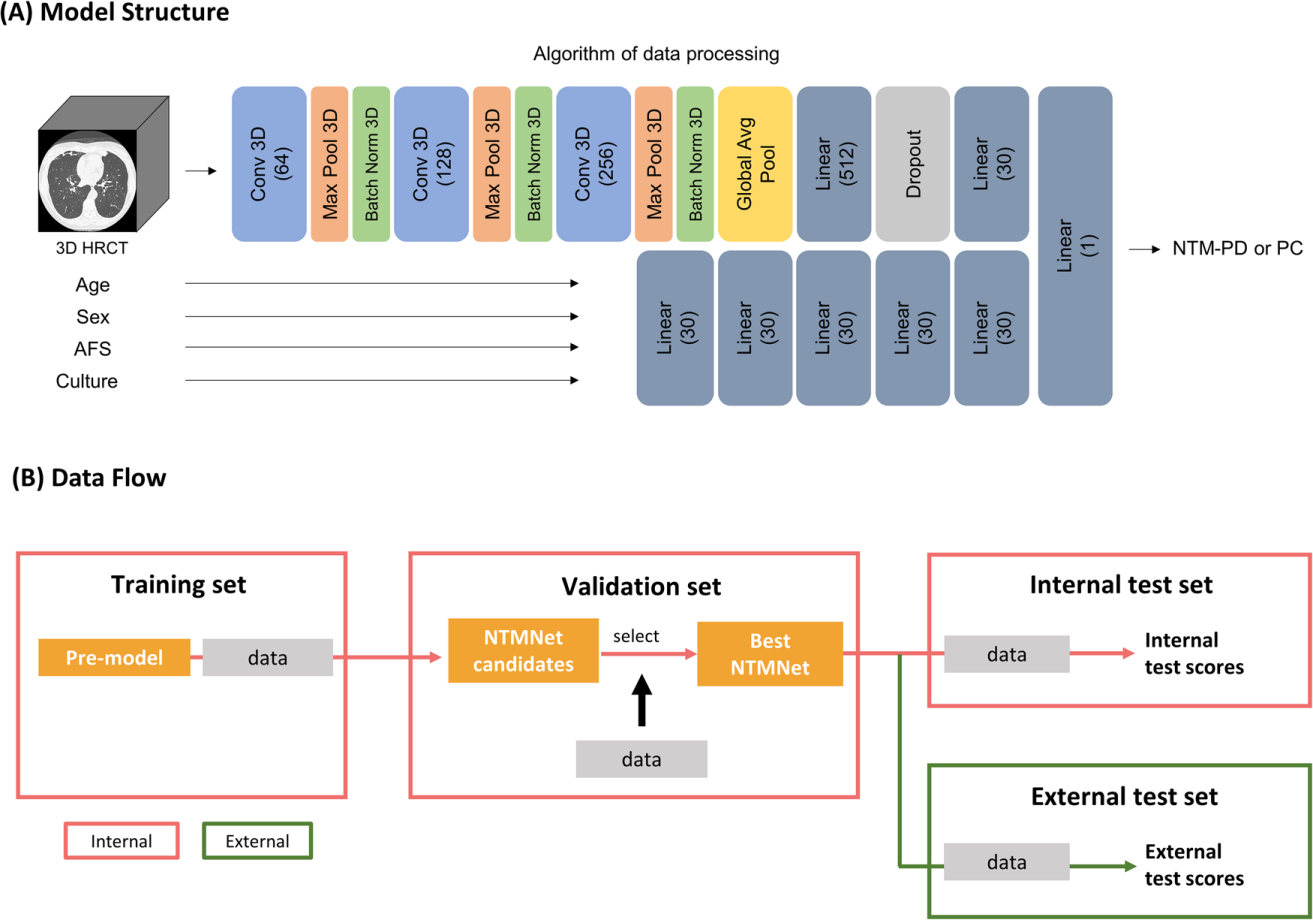
**A** Architecture of the NTMNet model. 3D HRCT, three-dimensional high-resolution computed tomography; conv, convolution; AFS, acid-fast smear; NTM-PD, nontuberculous mycobacterial pulmonary disease; NTM-PC, nontuberculous mycobacterial pulmonary colonization. **B** Development process of the NTMNet model and the data flow

Given that the determination of NTM disease status involves a comprehensive evaluation encompassing clinical, radiographic, and microbiologic criteria [4], we developed a multimodality deep learning model named NTMNet to access patient data across clinical, radiographic, and microbiologic domains. An overview of the structure of NTMNet, which comprises a three-dimensional convolutional neural network (3D-CNN) and a multilayer perceptron (MLP), is shown in Fig. 1A. Detailed information on the model structure is provided in Appendix B. The 3D-CNN model generated a value between 0 and 1 for each input chest CT, serving as an estimated probability of NTM disease status. Simultaneously, the MLP model utilized clinically significant variables, including age, sex, AFS, and NTM species, to predict NTM disease status [8, 9]. Finally, we merged the feature maps before the output layer of the 3D-CNN and MLP and passed them through a new fully connected layer to obtain the final prediction result of our NTMNet.

The model training process is illustrated in Fig. 1B. Following the training phase in the internal training set, the internal validation set was employed to assess the adequacy of the training results and to select the best-performing model.

### Evaluation of the NTMNet performance and reader study

The performance of NTMNet was evaluated using data from the internal test set, and the external test set was utilized to assess the external generalizability of the model, as illustrated in Fig. 1B. Additionally, the performance of the model within prespecified subgroups, including age, sex, smoking status, AFS positivity, NTM species and disease status, was evaluated. The assessment of the model’s prediction performance was based on the area under the receiver operating characteristic curve (AUC), accuracy, sensitivity, specificity, and F1 score. Calibration, depicted in a plot comparing predicted NTM disease status versus actual probability, was assessed using the bootstrap resampling method [14].

In parallel, we conducted a reader study to compare the performance of NTMNet with that of three board-certified pulmonologists with at least 5 years of clinical experience in managing NTM patients. Twenty patients from the external test set (11 with NTM-PC and 9 with NTM-PD) were randomly selected, and the pulmonologists independently assessed their disease status.

### Model visualization

To understand how our 3D-CNN model distinguished between NTM-PD and NTM-PC on chest CTs, we visualized the model’s attention using a combined approach of gradient-weighted class activation mapping [15] and integrated gradients [16]. In the evaluation of clinical variables using the MLP, we employed the Shapley additive explanation (SHAP) value to assess the importance of each variable [17].

### Statistical analysis

All the variables are presented as numbers (percentages) or means ± standard deviations as appropriate. Intergroup differences for continuous variables were analyzed using Student’s *t*-test or one-way analysis of variance (ANOVA), while the chi-square test was employed for categorical variables. All *p*-values were two-sided, and statistical significance was set at *p* < 0.05. The performance metrics with 95% confidence intervals (CIs) were calculated over 1000 bootstrap iterations. In each iteration, we resampled the data from the entire testing dataset and tested the model to obtain the results.

## Results

### Selection of study participants and dataset establishment

**Fig. 2:**
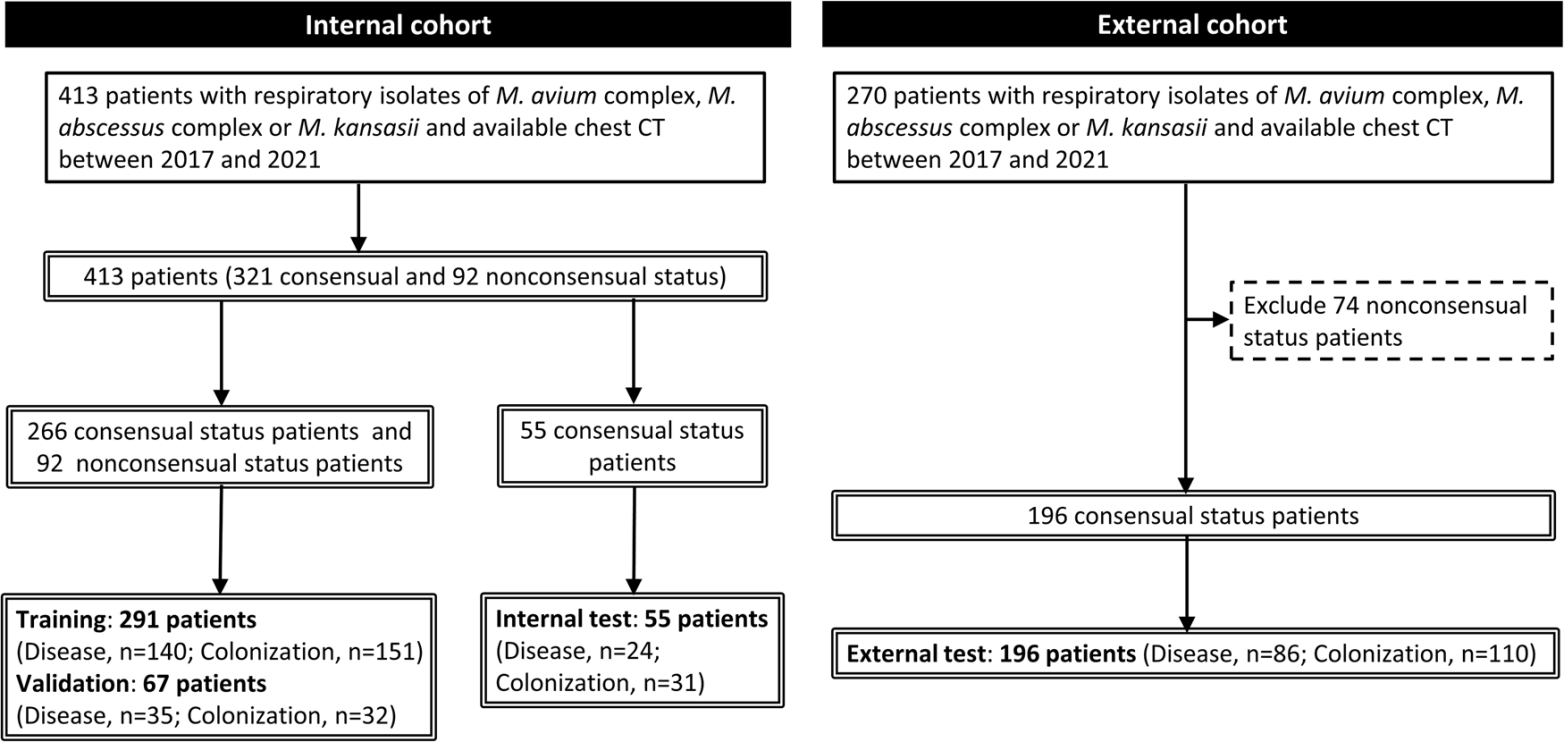
Flowchart of dataset establishment from patients with respiratory nontuberculous mycobacterial isolates

As shown in Fig. 2, initially, a total of 413 and 270 patients with respiratory NTM isolates were included in the internal and external cohorts, respectively. Three NTM experts independently assessed the NTM disease status of these patients, reaching a consensus in 517 patients (75.7%) (Appendix C). Compared with patients with a nonconsensual disease status, those with a consensus disease status were younger (67.8 ± 14.4 vs. 71.1 ± 13.0, *p* = 0.040) and exhibited a higher rate of cavitation on chest CT (13.3% vs. 4.3%, *p* = 0.014) (Appendix D).

After 74 patients in the external cohort whose disease status was nonconsensual were excluded, the final enrollment included 413 and 196 patients in the internal and external cohorts, respectively. Among them, the prevalence of NTM-PD was 46.8%. Subsequently, 291 and 67 patients were assigned to the internal training and validation sets, respectively. Furthermore, 55 and 196 patients with a consensus disease status were assigned to the internal and external test sets, respectively.

### Clinical characteristics of the enrolled patients

The clinical characteristics of the enrolled patients are summarized in Table 1.

**Table 1:** Demographics, microbiology and radiology data of patients with respiratory isolates of nontuberculous mycobacteria

	Internal cohort, training (*n* = 291)			Internal cohort, internal test (*n* = 55)			External cohort, external test (*n* = 196)		
	Colonization	Disease	*p*-value	Colonization	Disease	*p*-value	Colonization	Disease	*p*-value
	(*N* = 151)	(*n* = 140)		(*N* = 31)	(*n* = 24)		(*N* = 110)	(*n* = 86)	
Age (years)*	70.9 ± 13.0	68.9 ± 12.1	0.186	68.1 ± 15.1	66.5 ± 10.7	0.677	69.4 ± 15.7	63.1 ± 15.4	0.005
Female, *n* (%)*	81 (54%)	89 (64%)	0.086	18 (58%)	15 (63%)	0.739	38 (35%)	44 (51%)	0.019
Acid-fast smear			< 0.001			0.007			< 0.001
Negative	131 (87%)	76 (54%)		29 (94%)	14 (58%)		102 (93%)	47 (55%)	
Low-grade positive (1, 2)	13 (9%)	30 (21%)		1 (3%)	6 (25%)		7 (6%)	24 (28%)	
High-grade positive (3, 4)	7 (5%)	34 (24%)		1 (3%)	4 (17%)		1 (1%)	15 (17%)	
NTM species			0.776			0.552			0.006
* M. avium* complex*	91 (60%)	90 (64%)		19 (61%)	16 (67%)		53 (48%)	41 (48%)	
* M. abscessus* complex	46 (31%)	38 (27%)		11 (36%)	6 (25%)		43 (39%)	20 (23%)	
M. kansasii*	14 (9%)	12 (9%)		1 (3%)	2 (8%)		14 (13%)	25 (29%)	
Number of NTM isolates*	2.0 ± 2.2	6.5 ± 8.9	< 0.001	1.7 ± 1.6	12.0 ± 20.0	0.006	1.5 ± 1.0	3.4 ± 5.1	< 0.001
Chest CT pattern									
Fibrocalcified lesion	39 (26%)	22 (16%)	0.034	12 (39%)	2 (8%)	0.010	46 (42%)	7 (8%)	< 0.001
Nodule or mass*	47 (31%)	95 (68%)	< 0.001	6 (19%)	21 (88%)	< 0.001	11 (10%)	62 (72%)	< 0.001
Cavitation	2 (1%)	31 (22%)	< 0.001	1 (3%)	4 (17%)	0.086	1 (1%)	25 (29%)	< 0.001
Consolidation	53 (35%)	24 (17%)	0.001	9 (29%)	4 (17%)	0.284	29 (26%)	9 (11%)	0.005
Bronchiectasis	42 (28%)	103 (74%)	< 0.001	6 (19%)	19 (79%)	< 0.001	24 (22%)	63 (73%)	< 0.001
Pleural effusion*	26 (17%)	4 (3%)	< 0.001	4 (13%)	1 (4%)	0.373	4 (4%)	0 (0%)	0.132

*NTM* nontuberculous mycobacteria, *CT* computed tomography

* *p* < 0.05 compared among training, internal test sets of the internal cohort, and the external test set of the external cohort

When patients with NTM-PD were compared with those with NTM-PC, NTM-PD patients demonstrated significantly higher percentages of AFS positivity, 24% vs. 5% (*p* < 0.001), 17% vs. 1% (*p* = 0.007), and 17% vs. 1% (*p* < 0.001), in the internal training, internal test, and external test sets, respectively. They also had a greater number of NTM isolates, with mean values of 6.5 vs. 2.0 (*p* < 0.001), 12.0 vs. 1.7 (*p* = 0.006), and 3.4 vs. 1.5 (*p* < 0.001) across the same datasets.

With respect to radiographic patterns on chest CT, patients with NTM-PD were significantly more likely to have bronchiectasis (74% vs. 28% (*p* < 0.001), 79% vs. 19% (*p* < 0.001), and 73% vs. 22% (*p* < 0.001)) and nodules or masses (68% vs. 31% (*p* < 0.001), 88% vs. 19% (*p* < 0.001), and 72% vs. 10% (*p* < 0.001)) in the internal training, internal test, and external test sets, respectively. In contrast, patients with NTM-PCs were more likely to present with fibrocalcified lesions (26% vs. 16% (*p* = 0.034) and 42% vs. 8% (*p* < 0.001)) and consolidations (35% vs. 17% (*p* = 0.001) and 26% vs. 11% (*p* = 0.005)) in the internal training and external test sets, respectively.

### Performance of NTMNet

**Fig. 3:**
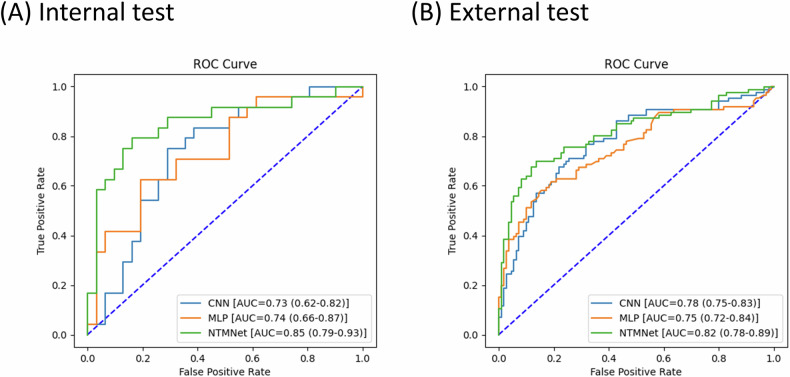
Area under the receiver operating characteristic curve (AUC) in both the internal (**A**) and external test (**B**) sets. CNN, convolutional neural network; MLP, multilayer perceptron

In the internal test set (Fig. 3A), the 3D-CNN model and MLP model achieved AUCs of 0.73 (95% CI: 0.62–0.82) and 0.74 (95% CI: 0.66–0.87), respectively, for predicting NTM disease status. The combined use of the 3D-CNN and MLP models, as represented by NTMNet, resulted in an AUC value of 0.85 (95% CI: 0.80–0.93). In the external test set (Fig. 3B), NTMNet achieved an AUC of 0.82 (95% CI: 0.78–0.89), whereas 3D-CNN performed at 0.78 (95% CI: 0.75–0.83) and MLP at 0.75 (95% CI: 0.72–0.84). The calibration plots of the NTMNet model in the internal and external test sets demonstrated good concordance between the model’s predicted and actual probabilities of NTM disease status (Appendix E).

### Subgroup analysis

**Fig. 4:**
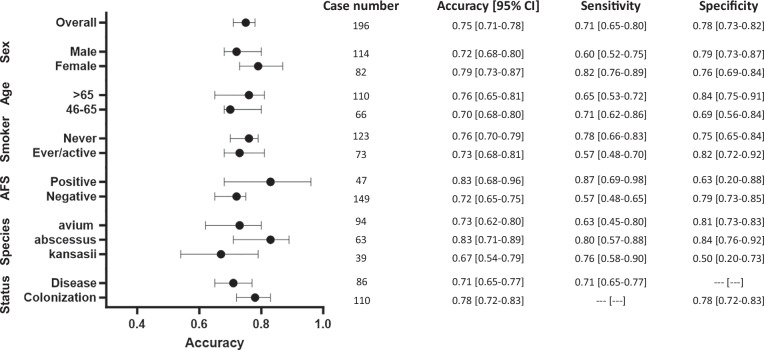
Subgroup analysis of the performance of the NTMNet model in the external test set. AFS, acid-fast smear

In the internal test set (Appendix F), NTMNet achieved satisfactory accuracy in terms of NTM disease status, ranging between 0.7 and 0.8 in most of the subgroups. Remarkably, NTMNet demonstrated superior predictive accuracy in patients with positive AFS than in those without (0.92 vs. 0.72, *p* < 0.001). In the external test set (Fig. 4 and Appendix G), NTMNet demonstrated a consistent predictive accuracy, maintaining a performance range between 0.7 and 0.8 in all subgroups except for patients with isolates of *Mycobacterium kansasii* (0.67). Further detailed data on the AUC, sensitivity, specificity, and F1 score are provided in Appendices F and G.

### Results of the reader study

In the reader study, NTMNet achieved a 75% accuracy in determining NTM disease status, which was statistically comparable to that of the three pulmonologists (85%, 95% and 65%) as shown in Appendix H.

### Model visualization

**Fig. 5:**
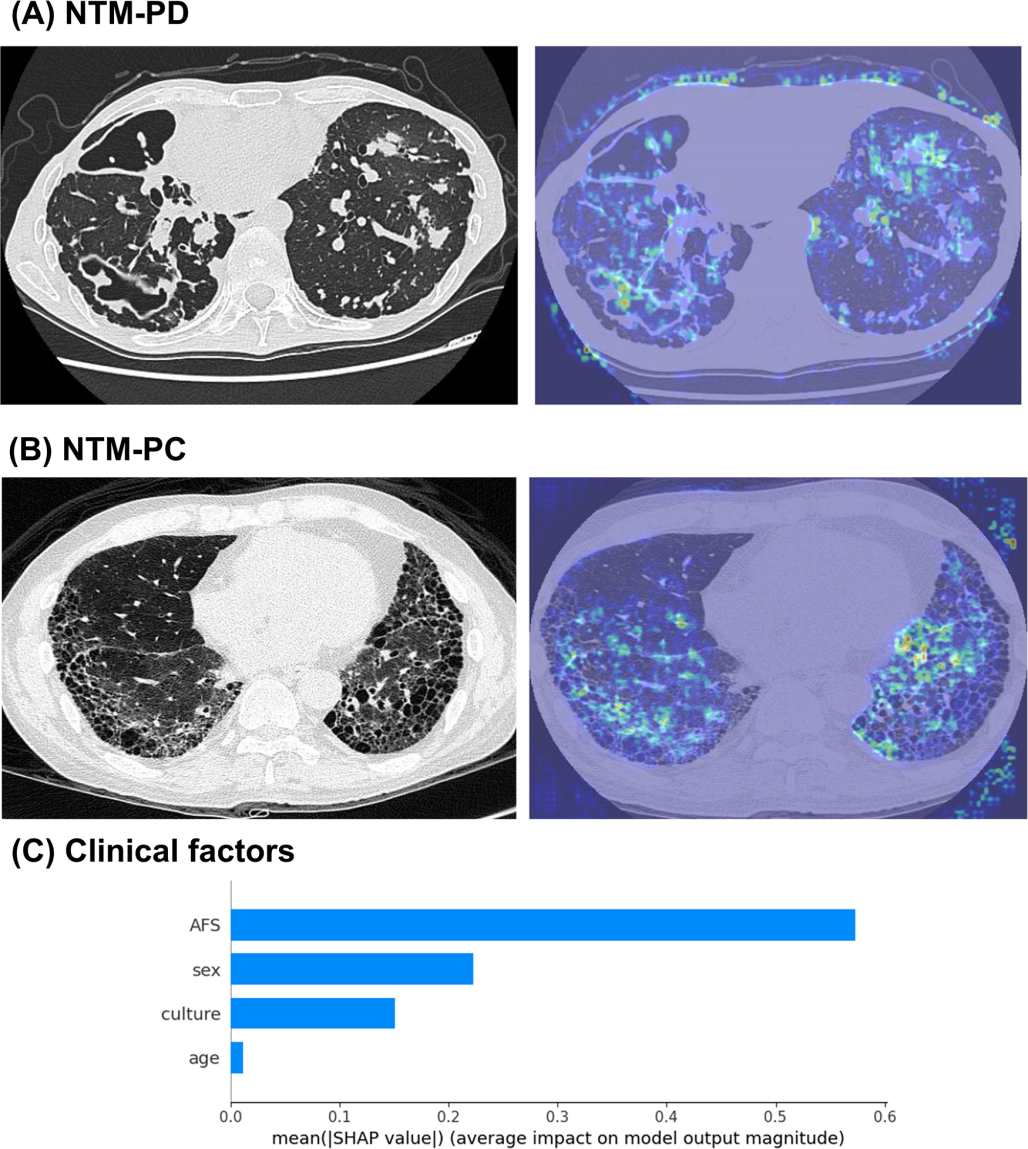
The class activation heatmaps for NTM-PD (**A**) and NTM-PC (**B**); the colors on the heatmap represent the diagnostic weights of determining the classification. The hotter the colors are (red and yellow), the more important the areas are to the final decision in the model. **C** The Shapley additive explanation (SHAP) value for assessing the importance of each clinical variable in disease status determination

Figure 5A shows a typical chest CT of a patient exhibiting cavities, bronchiectasis, and multiple nodules, which are characteristic radiographic features often observed in NTM-PD. Figure 5B shows a chest CT from a patient with idiopathic pulmonary fibrosis exhibiting severe fibrosis. In these representative cases, our 3D-CNN model accurately localized the lesions and classified the patients into NTM-PD and NTM-PC, respectively. As shown in Fig. 5C, the SHAP value indicates that AFS is the most important variable for the MLP when determining NTM disease status.

## Discussion

Our NTMNet model represents the first developed deep learning model that evaluates patients’ chest CT images and clinical information simultaneously, in alignment with the current NTM diagnostic guidelines [4], to distinguish between patients with NTM-PD and NTM-PC. Its classification performance was satisfactory and closely aligned with that of experienced pulmonologists. Furthermore, our model exhibited consistent diagnostic performance across different subgroups and disease prevalences, as indicated by subgroup analysis and calibration plots. These findings lay a robust foundation for the future role of deep learning-based models in assisting clinicians, including those without NTM expertise or in settings with limited access to specialists, to effectively differentiate NTM disease status among patients with respiratory NTM isolates.

Despite the clear criteria provided in the current NTM guideline for distinguishing between NTM-PD and NTM-PC [4], differentiation remains subjective and challenging for clinicians [18]. Expert consultation is often necessary [4, 19], yet diagnostic discrepancies persist even among specialists. In our study, a consensus on disease status was reached for only 75.7% of patients under retrospective expert review. This variability is likely even greater among nonexperts [20] or in cases with atypical or ambiguous radiographic findings, such as patterns other than fibrocavitary or nodular bronchiectatic. To address this, we employed a majority consensus approach among three NTM experts, although the possibility of misclassification cannot be entirely excluded in a retrospective setting.

While deep learning has been applied to distinguish NTM-PD from pulmonary TB [11, 21], no studies have yet used AI to differentiate NTM-PD from NTM-PC, an important but unmet clinical need. Some studies have attempted to offer a solution through alternative methods. Our previous study utilized breathomic analysis with an electronic nose and distinguished NTM-PD from NTM-PC, with AUCs ranging from 0.68 to 0.74 in a validation cohort [7]. Another study revealed soluble T-cell immunoglobulin mucin domain 3 as an independent factor for differentiating NTM-PD from NTM-PC, with an AUC of 0.68 [9]. In comparison, our NTMNet model achieved numerically higher AUCs of 0.85 and 0.82 in differentiating NTM-PD and NTM-PC within the internal and external test sets, respectively. This suggests its strong potential to support clinical decision-making using only routine imaging and clinical data, without the need for additional samples or invasive procedures.

A key strength of our NTMNet model is the incorporation of chest CT images during training, which enhances its accuracy and reliability. Compared with CXR, chest CT is more effective at detecting NTM-PD-related lesions such as centrilobular nodules, tree-in-bud lesions, and bronchiectasis. Consequently, the current NTM diagnostic guideline recommends performing chest CT in individuals suspected of having NTM-PD [22]. Our NTMNet model demonstrated robust and consistent performance across training, internal, and external test sets with similar microbiological and radiological profiles, supporting its generalizability under well-matched conditions. However, the slight decrease in performance in the external test set may reflect differences in CT acquisition protocols, image quality, and local disease prevalence [23]. Variations in slice thickness, reconstruction methods, and image noise can affect feature extraction, whereas regional epidemiology influences predictive utility. Therefore, onsite validation and potential fine-tuning are essential before clinical implementation to ensure optimal accuracy and reliability of the model in local settings.

Furthermore, we explored the classification rationale within the NTMNet model using visualization heatmaps and SHAP values. While Grad-CAM provides a visual approximation of the regions most strongly influencing the model’s prediction, its interpretability is inherently limited. This method produces coarse, low-resolution heatmaps that may not align precisely with anatomical structures, potentially leading to ambiguous localization. Moreover, Grad-CAM highlights correlations rather than causal relationships, meaning that the visualized regions may not directly represent features critical to the decision. However, Fig. 5 shows that the heatmaps highlight cavities, bronchiectasis, and multiple nodules as key imaging features contributing to the classification of NTM disease status. SHAP value analysis revealed that AFS was the most influential variable in determining NTM disease status. These findings are consistent with the radiographic criteria outlined in the current NTM guideline [4] and align with a previous study reporting that positive AFS is associated with NTM-PD [24].

Our NTMNet model demonstrated consistent performance across datasets and subgroups, except for patients with isolates of *Mycobacterium kansasii* in the external test set. The exact reason behind this observation is unclear, but it may be attributed to the small sample size of patients with *Mycobacterium kansasii* isolation in the training cohort [25]. Future studies could leverage transfer learning from pretrained foundation models to improve the model’s performance in this subgroup. Calibration plots further confirmed the model’s stability across different NTM-PD prevalence levels, supporting its generalizability.

CNNs are widely regarded as the most effective AI models for disease detection in medical imaging because of their interpretability and superior image recognition capabilities. In contrast to existing approaches that predominantly utilize 2D-CNNs for disease analysis or prediction across various domains, our NTMNet is built on a 3D-CNN framework, setting it apart from previous studies [26, 27]. While 2D-CNNs have been applied to both 2D and 3D medical images by converting 3D images into a series of 2D slices for analysis [28–30], this method, despite being simpler and quicker to train, often sacrifices the interslice information crucial for comprehensive analysis. Studies have shown that the use of the full volumetric data of 3D images can improve performance compared with methods that analyze these images slice-by-slice [31–33]. The key advantage of this approach is the preservation of interslice information [34], which is crucial for maintaining the contextual relationships between CT slices. This insight is the foundation for our decision to use a 3D-CNN architecture in the development of NTMNet, with the goal of enabling more natural and effective learning of the distribution patterns among CT scans in our dataset.

Our study has several limitations. First, clinical symptoms were not included in the development of the NTMNet because of their ambiguity and inconsistency in reporting, making them unreliable variables. Second, we excluded patients with a nonconsensual disease status in the internal and external tests to ensure diagnostic clarity during model performance evaluation. While this approach enhanced the reliability of our results, it may also have limited the generalizability of the model to patients with ambiguous disease status. In our cohort, approximately 25% of patients with respiratory NTM isolates had nonconsensual diagnoses; these individuals tended to be older and exhibited a lower prevalence of cavitary lesions on chest CT. Although patients whose status was ambiguous were included during model training and validation, the performance of the NTMNet model in this subgroup warrants careful assessment in real-world settings.

In conclusion, our NTMNet model, which uses 3D-CNN and incorporates chest CT and clinical information, exhibited satisfactory performance in distinguishing disease status among patients with respiratory NTM isolates. These results suggest that deep learning-based models may have the potential to enhance the diagnostic performance of clinical physicians, potentially reaching the level of experienced pulmonologists.

## Supplementary information

ELECTRONIC SUPPLEMENTARY MATERIAL

## Data Availability

The datasets used and/or analyzed during the current study are available from the corresponding author on reasonable request.

## Abbreviations

3D-CNN: Three-dimensional convolutional neural network
AFS: Acid-fast smear
AI: Artificial intelligence
AUC: Area under the curve
CT: Computed tomography
CXR: Chest X-ray
MLP: Multilayer perceptron
NTM: Nontuberculous mycobacteria
NTM-PC: NTM pulmonary colonization
NTM-PD: NTM pulmonary disease
SHAP: Shapley additive explanation
TB: Tuberculosis
