# Supplementary material for: AI-assisted differentiation of nontuberculous mycobacterial pulmonary disease from colonization: a multi-center study
Source: Insights Imaging. 2025 Nov 9;16:249. doi: 10.1186/s13244-025-02131-1 (PMC12597856; doi:10.1186/s13244-025-02131-1)
Supplement: Supplementary file 1 — ELECTRONIC SUPPLEMENTARY MATERIAL [file 13244_2025_2131_MOESM1_ESM.pdf]

AI-Assisted Differentiation of Nontuberculous Mycobacterial Pulmonary Disease from Colonization: A Multi-Center Study

ELECTRONIC SUPPLEMENTARY MATERIAL

Appendix A. Processing for chest CT image

The raw two-dimensional (2D) chest CT slices of a patient need to be preprocessed to lower the bias between cases and concatenated as three-dimensional (3D) images. We first normalize each 2D chest CT slice, concatenate them as 3D images, and apply Spline interpolated zoom (SIZ) for a patient. To increase the diversity of training data, we use random rotation and random gamma correction as the augmentation methods. Besides, we apply 2 additional pre-processing methods to our dataset: Global Brightness Normalization (GBN) and alignment. GBN will let the average brightness of CT scans among different patients be the same. The whole 3D CT scans of a patient case will be adjusted to make the average brightness value of it becomes the target brightness value that we desire. Since CT scans might be captured by different views in our dataset, so we designed an alignment method to let the view of CT scans among different patients be the same.

There are mainly 3 steps in the alignment process: 1) First, several slices in the middle among all slices of a patient will be selected. 2) Then, we will try to find a minimal circle that can cover the whole lung segmentation among the selected middle slices. 3) Finally, the circle will be extended as a cylinder along the craniocaudal axis, and we will use the cylinder to cut the new view of the 3D CT scans volume.

Appendix B. NTMNet model structure

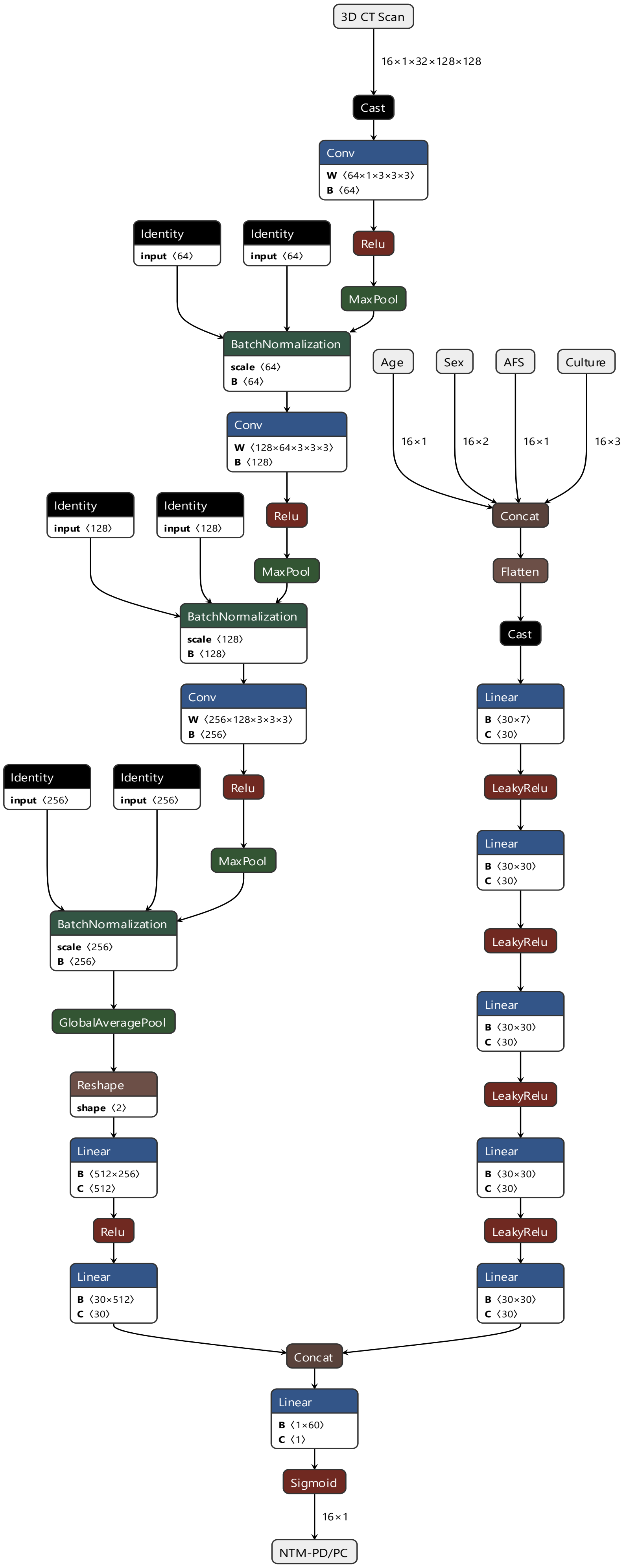

Appendix C. The NTM disease status determination by three clinical NTM experts

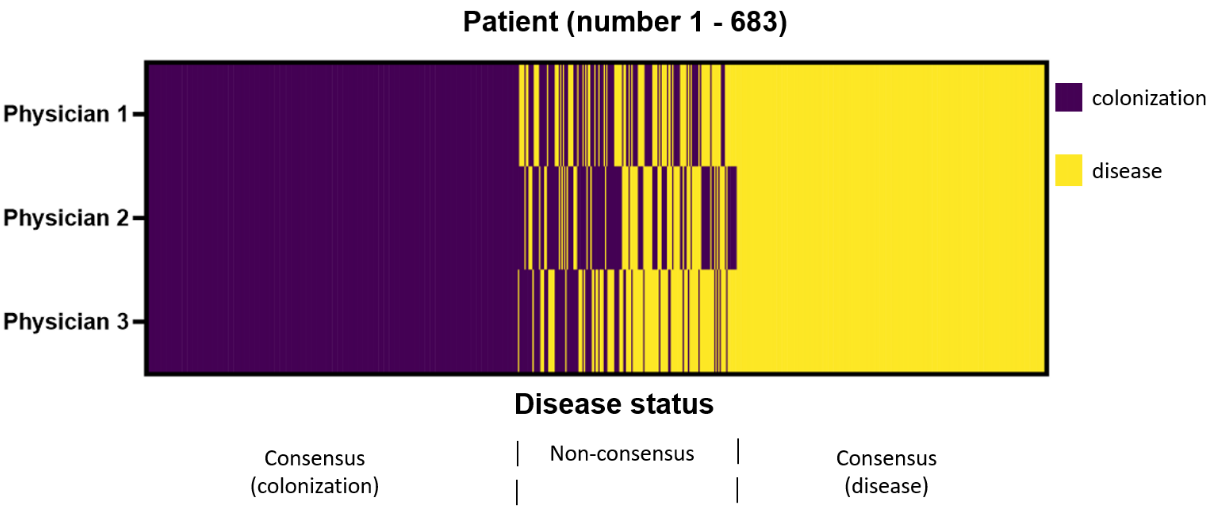

Figure. The heatmap illustrates the determination of NTM disease status among three clinical NTM experts. Each column represents a patient. The columns highlighted in yellow denote cases labeled as disease by the physicians, while the columns in purple indicate cases labeled as colonization by the physicians.

Appendix D. The clinical characteristics of patients with consensual and nonconsensual disease status

	Consensus (n=517)	Non-Consensus (n=92)	*P*
Age (years)	67.8 ± 14.4	71.1 ± 13.0	0.040
Female, n (%)	281 (54.4%)	45 (48.9%)	0.335
**Acid-fast smear**			0.918
Negative	373 (72.1%)	67 (72.8%)	
Low-grade positive (1, 2)	81 (15.7%)	13 (14.1%)	
High-grade positive (3, 4)	63 (12.2%)	12 (13.0%)	
**NTM species**			0.566
M. avium complex	295 (57.1%)	47 (51.1%)	
M. abscessus complex	164 (31.7%)	33 (35.9%)	
M. kansasii	58 (11.2%)	12 (13.0%)	
**Chest CT pattern**			
Fibrocalcified lesion	127 (24.6%)	18 (19.6%)	0.300
Nodule or mass	228 (44.1%)	46 (50.0%)	0.295
Cavitation	69 (13.3%)	4 (4.3%)	0.014
Consolidation	117 (22.6%)	28 (30.4%)	0.105
Bronchiectasis	244 (47.2%)	48 (52.2%)	0.378
Pleural effusion	38 (7.4%)	6 (6.5%)	0.777

Abbreviation: NTM, nontuberculous mycobacteria; CT, computed tomography;

Appendix E. Calibration plots of the NTMNet

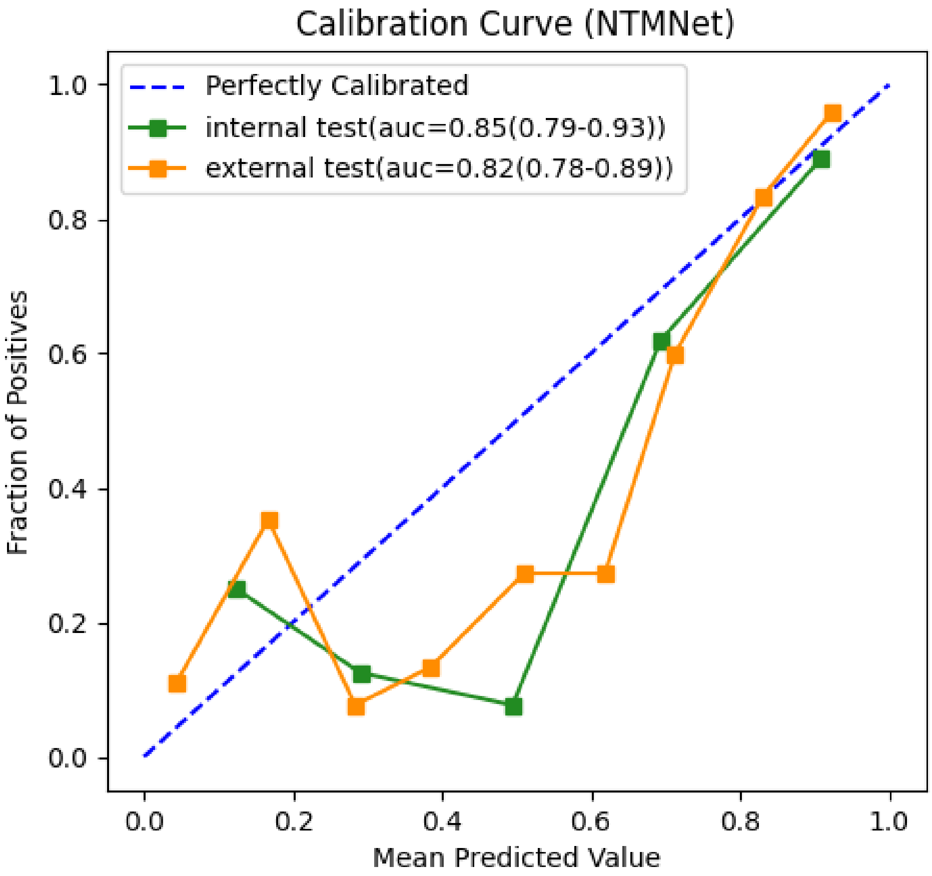

Figure. Calibration plots were generated to illustrate the NTMNet model's predicted and actual probability of NTM disease status in both the internal and external test sets.

Appendix F. Diagnostic performance of each subgroup in the internal test set

	Internal test				
	**AUC**	**Accuracy**	**Sensitivity**	**Specificity**	**F1-score**
**Overall**	0.85 [0.80-0.93]	0.76 [0.67-0.87]	0.79 [0.62-0.92]	0.74 [0.56-0.90]	0.75 [0.58-0.86]
**Sex**					
Male	0.81 [0.66-0.90]	0.77 [0.68-0.86]	0.67 [0.25-0.86]	0.85 [0.71-1.00]	0.71 [0.36-0.80]
Female	0.88 [0.82-0.93]	0.76 [0.67-0.79]	0.87 [0.82-1.00]	0.67 [0.40-0.76]	0.76 [0.69-0.83]
**Age**					
> 65	0.92 [0.84-0.99]	0.83 [0.74-0.94]	0.93 [0.83-1.00]	0.75 [0.68-0.96]	0.82 [0.71-0.90]
46-65	0.75 [0.55-0.98]	0.63 [0.44-0.81]	0.63 [0.29-1.00]	0.63 [0.43-0.89]	0.63 [0.33-0.80]
**Smoker**					
Never	0.86 [0.74-0.92]	0.77 [0.58-0.88]	0.81 [0.62-0.90]	0.74 [0.48-0.86]	0.76 [0.64-0.89]
Ever/active	0.83 [0.40-1.00]	0.71 [0.43-0.86]	0.67 [0.00-1.00]	0.75 [0.40-1.00]	0.67 [0.00-0.91]
**AFS**					
Positive	0.70 [0.00-1.00]	0.92 [0.92-1.00]	1.00 [1.00-1.00]	0.50 [0.00-1.00]	0.95 [0.95-1.00]
Negative	0.80 [0.59-0.85]	0.72 [0.56-0.77]	0.64 [0.46-0.80]	0.76 [0.60-0.88]	0.60 [0.39-0.74]
**NTM species**					
avium	0.77 [0.58-0.90]	0.71 [0.63-0.86]	0.75 [0.67-0.88]	0.68 [0.61-0.88]	0.71 [0.55-0.88]
abscessus	0.97 [0.89-1.00]	0.82 [0.65-0.88]	0.83 [0.56-1.00]	0.82 [0.56-1.00]	0.77 [0.60-0.83]
kansasii	1.00 [0.00-1.00]	1.00 [1.00-1.00]	1.00 [1.00-1.00]	1.00 [0.00-1.00]	1.00 [1.00-1.00]
**NTM status**					
Disease	1.00 [0.00-1.00]	0.79 [0.75-0.88]	0.79 [0.75-0.88]	--- [---]	0.88 [0.86-0.93]
Colonization	1.00 [0.00-1.00]	0.74 [0.58-0.84]	--- [---]	0.74 [0.58-0.84]	--- [---]

Abbreviation: AUC, area under the receiver operating characteristic curve; AFS, acid-fast smear; NTM, nontuberculous mycobacteria;

Appendix G. Diagnostic performance of each subgroup in the external test set

	External test				
	AUC	Accuracy	Sensitivity	Specificity	F1-score
**Overall**	0.82 [0.78-0.89]	0.75 [0.71-0.78]	0.71 [0.65-0.80]	0.78 [0.73-0.82]	0.71 [0.66-0.76]
**Sex**					
Male	0.74 [0.68-0.80]	0.72 [0.68-0.76]	0.60 [0.52-0.75]	0.79 [0.73-0.87]	0.61 [0.55-0.69]
Female	0.88 [0.85-0.93]	0.79 [0.73-0.87]	0.82 [0.76-0.89]	0.76 [0.69-0.84]	0.81 [0.73-0.88]
**Age**					
> 65	0.79 [0.66-0.84]	0.76 [0.65-0.81]	0.65 [0.53-0.72]	0.84 [0.75-0.91]	0.68 [0.55-0.74]
46-65	0.77 [0.77-0.86]	0.70 [0.68-0.80]	0.71 [0.62-0.86]	0.69 [0.56-0.84]	0.69 [0.59-0.78]
**Smoker**					
Never	0.86 [0.80-0.90]	0.76 [0.70-0.79]	0.78 [0.66-0.83]	0.75 [0.65-0.84]	0.76 [0.67-0.80]
Ever/active	0.72 [0.60-0.84]	0.73 [0.68-0.81]	0.57 [0.48-0.70]	0.82 [0.72-0.92]	0.62 [0.55-0.70]
**AFS**					
Positive	0.80 [0.65-0.98]	0.83 [0.68-0.96]	0.87 [0.69-0.98]	0.63 [0.20-0.88]	0.89 [0.77-0.98]
Negative	0.76 [0.67-0.81]	0.72 [0.65-0.75]	0.57 [0.48-0.65]	0.79 [0.73-0.85]	0.57 [0.49-0.65]
**NTM species**					
avium	0.81 [0.70-0.87]	0.73 [0.62-0.80]	0.63 [0.45-0.80]	0.81 [0.73-0.83]	0.68 [0.54-0.80]
abscessus	0.87 [0.77-0.92]	0.83 [0.71-0.89]	0.80 [0.57-0.88]	0.84 [0.76-0.92]	0.74 [0.59-0.84]
kansasii	0.68 [0.54-0.88]	0.67 [0.54-0.79]	0.76 [0.58-0.90]	0.50 [0.20-0.73]	0.75 [0.65-0.87]
**NTM status**					
Disease	1.00 [0.00-1.00]	0.71 [0.65-0.77]	0.71 [0.65-0.77]	--- [---]	0.83 [0.79-0.87]
Colonization	1.00 [0.00-1.00]	0.78 [0.72-0.83]	--- [---]	0.78 [0.72-0.83]	--- [---]

Abbreviation: AUC, area under the receiver operating characteristic curve; AFS, acid-fast smear; NTM, nontuberculous mycobacteria;

Appendix H. Reader test

	11 NTM-PC and 9 NTM-PD in the external test set			
	**NTMNet**	**Pulmonologist 1**	**Pulmonologist 2**	**Pulmonologist 3**
**Accuracy**	75.0%	85.0%	95.0%	65.0%
P*		0.330	0.428	0.494

Abbreviation: NTM, nontuberculous mycobacteria; PC, pulmonary colonization; PD, pulmonary disease

P* compared between each pulmonologist and NTMNet on diagnosis accuracy.
